# Antimicrobial activity of indole-based melatonin analogues

**DOI:** 10.1186/1753-6561-6-S4-P27

**Published:** 2012-07-09

**Authors:** H Shirinzadeh, AD Yılmaz, N Yücel, N Altanlar, S Suzen, S Ozden

**Affiliations:** 1 Department of Pharmaceutical Chemistry, Faculty of Pharmacy, Ankara University, Turkey; 2 Department of Pharmaceutical Microbiology, Faculty of Pharmacy, Ankara University, Turkey; 3 Department of Microbiology, Gazi University, Faculty of Arts of Sciences, Ankara, Turkey

## Introduction

Methicillin-Resistant *Staphylococcus aureus* (MRSA) and Vancomycin-Resistant *Enterococcus* (VRE) have accomplished important rates of colonization and infection in most intensive care units. These multidrug-resistant strains of MRSA and VRE have been causing serious problems in health care. The rising clinical importance of drug-resistant pathogens has lent necessity to drug development research. In recent years, many 1*H*-indole derivatives including also Schiff base structures have been reported to exhibit chemotherapeutic properties such as antiviral, anti-tuberculosis, antifungal, and antibacterial activity. Hydrazone-type compounds containing an azomethine also represent a significant class of compounds for new drug development. The hydrazone group in molecules has an essential role in antimicrobial activity. It has been claimed that a number of hydrazide-hydrazone derivatives possess interesting antibacterial-antifungal and antituberculosis activities.

## Methods

A series of indole-3-aldehyde and 5-bromoindole-3-aldehyde hydrazide and hydrazones was evaluated for their in vitro antimicrobial activities using the 2-fold serial dilution technique against *S. aureus*, MRSA, *E. coli*, *B. subtilis* and *C. albicans*. Compounds that have halogenated phenyl ring, displayed better activity against MSRA and significant activity against *S. aureus* relative to ampicillin. As a part of our ongoing study nineteen indole hydrazone derivatives were tested for antibacterial activity using the 2-fold serial dilution technique.

## Results

The structure-activity relationships of the investigated indol hydrazone derivatives displayed that the aromaticity appeared to be significant for the antimicrobial activity. Generally, the activity of compounds was increased with the introduction of halogens in to the phenyl side chain. In this study compounds (2,4-difluoro), (2,5-difluoro), (3,5-difluoro), (3,5-dichloro), which have two “F” and two “Cl” atoms on the phenyl ring were found to be the most potent antimicrobial agents. Furthermore monohalogenated derivatives followed the dihalogenated compounds.

## Conclusions

In the present study it was thought worthwhile to investigate combined two potential pharmacophores in a single medium and to evaluate them for their synergistic antimicrobial activity. These results indicating that the halogen atom plays an important role in the antimicrobial activity of Schiff bases. As a result, further studies are needed to better understand the efficacy of indole-3-aldehyde hydrazide-hydrazones for the development of new antimicrobial agents.

